# A geographical location prediction method based on continuous time series Markov model

**DOI:** 10.1371/journal.pone.0207063

**Published:** 2018-11-19

**Authors:** Yongping Du, Chencheng Wang, Yanlei Qiao, Dongyue Zhao, Wenyang Guo

**Affiliations:** Faculty of Information Technology, Beijing University of Technology, Beijing, China; Liverpool John Moores University, UNITED KINGDOM

## Abstract

Trajectory data uploaded by mobile devices is growing quickly. It represents the movement of an individual or a device based on the longitude and latitude coordinates collected by GPS. The location based service has a broad application prospect in the real world. As the traditional location prediction models which are based on the discrete state sequence cannot predict the locations in real time, we propose a Continuous Time Series Markov Model (CTS-MM) to solve this problem. The method takes the Gaussian Mixed Model (GMM) to simulate the posterior probability of a location in the continuous time series. The probability calculation method and state transition model of the Hidden Markov Model (HMM) are improved to get the precise location prediction. The experimental results on GeoLife data show that CTS-MM performs better for location prediction in exact minute than traditional location prediction models.

## Introduction

Location-based Service (LBS) is a kind of information service that provides users geographical positions located by mobile devices and wireless network. There exists wealth information in the location data, such as user’s interests, user’s hobbies and user’s behavior pattern. LBS may be employed in a number of applications, including: location-based advertising [1], personalized weather services, entertainment [2], personal life and so on. An effective location prediction or recommendation can make the users have good experience.

The advances in location-acquisition and mobile communication technologies empower people to use location data with existing online social networks in a variety of ways. People can share their present location, record travel routes with GPS to share travel experiences in GeoLife [3]. Zheng [4] gives the overview of trajectory data mining, including the trajectory data preprocessing, pattern mining and classification, it explores the connections, correlations and differences among these existing techniques and also some public trajectory datasets are presented. Zheng [5] puts forward the approach to find the top-k candidate trips within the uncertain trajectory data. The historical data is used to inference the travel trip and it reduces the uncertainty of the user’s trajectory.

In order to improve the location service experience, it is needed to know the user’s location in advance. For example, if it can be predicted that the user will appear location B at 6:00pm based on the previous locations visited, the LBS provider can send the recommendation information or advertisements for restaurants in location B to the user in advance. Xue [6] puts forward the SubSyn algorithm for location prediction. The user’s historical trajectory data is decomposed into the set of sub trajectory, which increases the number of tracks and the training data size, and the prediction performance is improved. As for the track prediction, the method of Markov Model [7,8] is widely used and its central idea is to build the Markov chain for speculation. The algorithm of SMLP (Social-aware Mobile user Location Prediction algorithm) is put forward by Yu [9]. It integrates the user’s correlation with Markov Model for location prediction. Although the algorithm requires less space than the Markov model, the prediction results are heavily affected by the region division. Lian [10] proposes the CEPR(Collaborative Exploration and Periodically Returning model) algorithm which adopts collaborative filtering technique and the user historical behavior is used for location prediction and recommendation. They also give the correlation analysis [11] between the user statistical information and location predictability on the data of Gowalla(https://snap.stanford.edu/data/loc-gowalla.html). The prefix tree and heuristic search strategy are adopted to implement the personalized trip recommendation by Zhang [12]. Wu [13] uses Markov Random Field to predict the annotation of location records and the user’s destiny, the better performance is achieved when there exists more user’s records. Nghia [14] uses matrix factorization to select features and predicts the user’s location. Although the algorithm can predict geographic location on real time, their dataset is composed of tweets containing lots of semantic information. The results are influenced by people’s subjective emotions and expressions.

Gambs [15] extends a mobility model called Mobility Markov Chain (MMC) to incorporate the *n* previous visited locations for next location prediction. However, it cannot predict location within any time interval. Mathew [16]presents a hybrid method for predicting human mobility on the basis of Hidden Markov Models (HMMs). They use forward algorithm to compute the probability of possible sequences and return the next place from the sequence with the highest probability. But the experimental results on GeoLife is not satisfied with the highest Precision@5 of 26.40. Qiao [17] proposes a hybrid Markov based prediction model that contains three stages: mobility pattern discovery, variable-order Markov predictor and mobility pattern based users similarity calculation. The human trajectory data is extracted from data traffic of an LTE(Long Term Evolution) network. The extensive experimental evaluations should be conducted to compare with other related work on different datasets. Huang [18] proposes a predictive model taking into account activity changes. It is implemented for two users selected from the GeoLife dataset and gets performance improvement. The study results are limited by the spatial and temporal coverage of the dataset used and it should be applied to predict human movement by different days of the week with better quality data.

In addition, many other approaches are also used to build the prediction model, such as the association rules based method [19] and so on. However, all of these existing strategies cannot give the prediction based on the real time.

GeoLife(https://www.microsoft.com/en-us/download/details.aspx?id=52367) is the commonly used data for location based service, which records a broad range of users’ outdoor movements, including not only life routines but also some entertainments and sports activities. This trajectory dataset can be used in many research fields, such as mobility pattern mining, user activity recognition, location-based social networks and location recommendation.

In this paper, we address the issue of predicting the user’s location on the continuous time series based on the historical trajectory data and give the improvement to the original Markov model. The discrete time sequence is simulated to the continuous sequence by Gaussian Mixture Model.

## Potential location discovery method

### Location prediction structure

**Fig 1:**
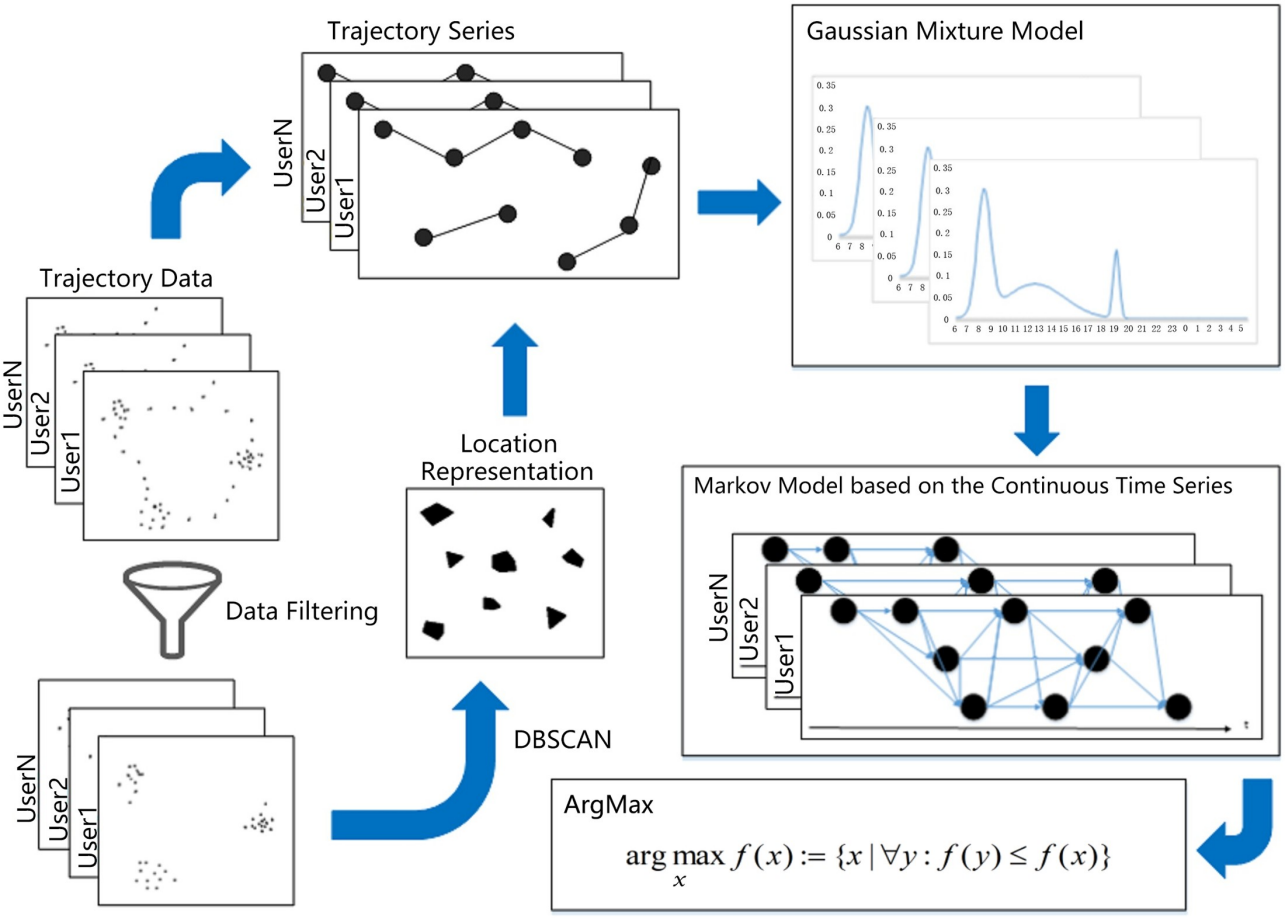
Location prediction based on the real time series.

The potential location is discovered by filtering and clustering technique on large scale tracing point data. The structure of location prediction based on the real time series is shown in Fig 1.

The user’s trajectory data, which is represented by <Time, Location> series, is filtered and clustered to produce a series of candidate location. Gaussian mixture modeling is implemented on the serial density of each location. Combined with the transition probability matrix, the original markov model is improved to the new model which is based on the continuous time series. The symbols used in this paper are shown in Table 1.

**Table 1:** The symbol definition.

Symbol	Definition
T	The continuous trajectory data series with a starting tracing point and an ending tracing point. $T_{j}^{i}$ denotes the *jth* tracing point within the *ith* trajectory.
*L*	The location area with a large number of tracing points by clustering, such as business area and park. *L*_*i*_ denotes the *ith* Location.
*T*	The set of user’s sign-in time.
Υ	The set of time for the possible location transition.
∂	Threshold for the walking time.
$I_{k}^{i,j}$	The *kth* trajectory from location *L*_*i*_ to *L*_*j*_.
*M*	Probability matrix for location transition.
*ξ*	Threshold for the location transition time.

### Tracing data filtering

The noise data should be filtered to reduce the interference. The filtering algorithm retains the tracing points which maybe selected as the candidate location and discards the irrelevant tracing points. Three kinds of filtering strategies are shown in the following.

**Fig 2:**
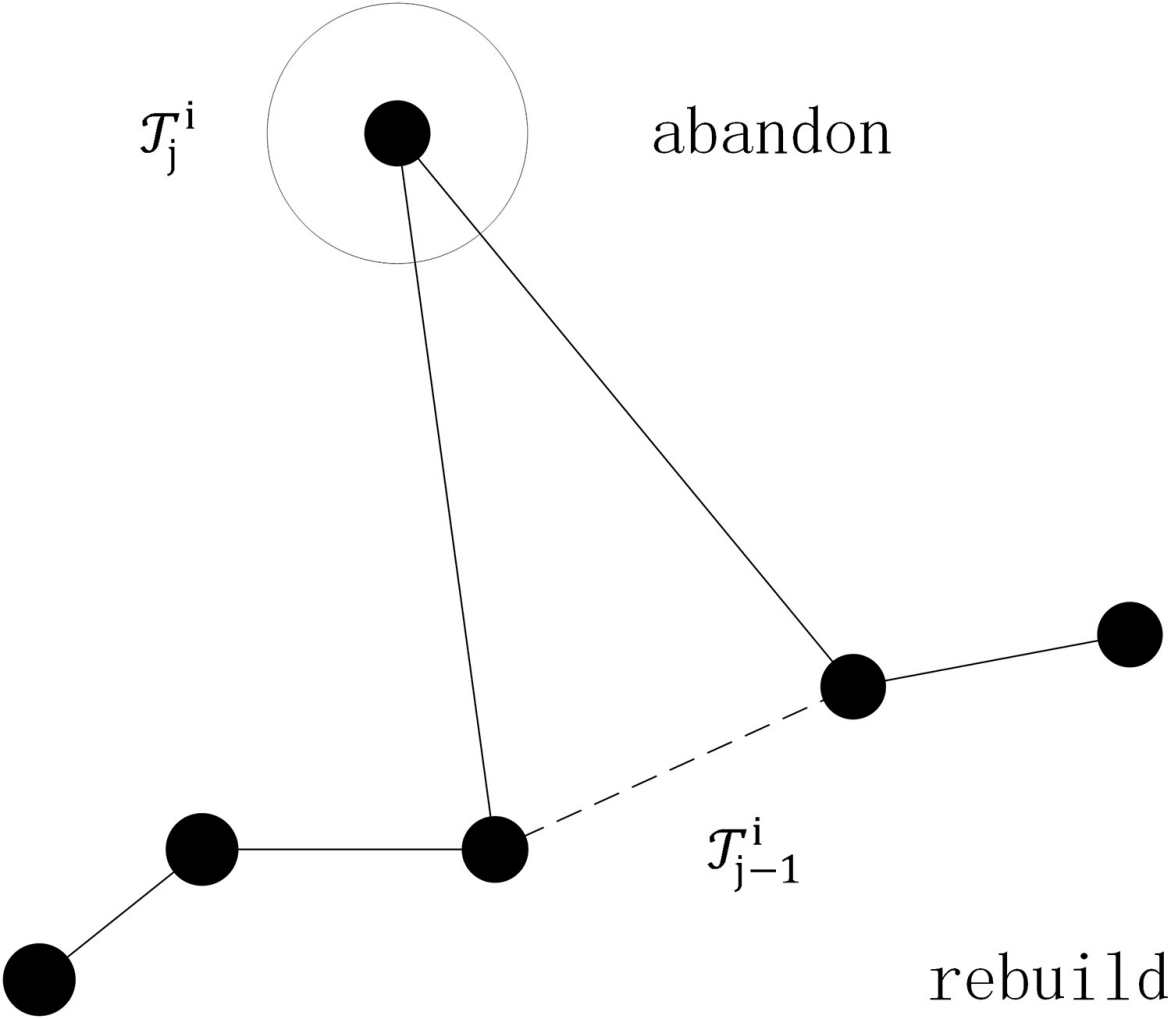
The drift tracing point filtering.

1. Filtering the drift tracing point and rebuild the trajectory. The sample is shown in Fig 2.
  The drifting phenomenon is caused by the strength of GPS signal and the satellite switching. The filtering rule is shown in the following:
  ***Rule 1*.** For $T_{j}^{i}\in T^{i},(j\;=\;1,2\;\ldots \;N)$, if ${|T}_{j}^{i}{\;T}_{j-1}^{i}|>\xi ,\;$ then delete $T_{j}^{i}$ in $T^{i}$.
  Here, ${|T}_{j}^{i}{\;T}_{j-1}^{i}|$ denotes the distance between tracing point $T_{j}^{i}$ and $T_{j-1}^{i}$. *ξ* is the threshold.
2. Filtering the tracing point with a higher speed than the average walking speed.
  The pedestrian speed is relatively slow usually in the candidate location where the traffic is greater than the regional carrying capacity. On the other hand, there exists the activity which can only be carried at lower speed, such as playing and watching.
  For $T_{j}^{i}\in T^{i},(j\;=\;1,2\;\ldots \;N-1)$, the average speed *v*_*j*_ is computed by formula 1.
  $$v_{j}=\frac{\frac{|T_{j-1}^{i}\;T_{j}^{i}{|}^{2}}{\Delta t_{j-1,j}}+\frac{|T_{j}^{i}\;T_{j+1}^{i}{|}^{2}}{\Delta t_{j,j+1}}}{|T_{j-1}^{i}\;T_{j}^{i}\left|+\;|T_{j}^{i}\;T_{j+1}^{i}\right|}$$ (1)
  The filtering rule is:
  ***Rule 2*.** if (*v*_*j*_ > *η*), then discard the tracing points ${{\;T}_{j-1}^{i},T}_{j}^{i},{\;T}_{j+1}^{i}$ in $T^{i}$.
  Here, *η* value is set to 1.5m/s which is faster than the average walking speed.
3. Filtering the trajectory with shorter residence time.
  The trajectory with shorter residence time has a little chance to be the part of the candidate location. The filtering rule:
  ***Rule 3*.** if ${\Delta t}_{0,N}(T^{i})<\partial$, then delete $T^{i}$.
  Here, N denotes the tracing point number in trajectory $T^{i}$ and ${\Delta t}_{0,N}(T^{i})$ represents the residence time for trajectory $T^{i}$. The threshold *∂* value is set to 20 minutes which is also used by Huang [18] and Zheng [20] to extract meaningful human activities.

After filtering the noisy tracing point, we adopt the DBSCAN clustering algorithm to discover the candidate location. K-means and DBSCAN are the most commonly used algorithms for location clustering. However, K-means algorithm needs to set the number of clusters in advance, and it is not suitable for the experimental dataset GeoLife with the larger size and dispersion. We also try the Birch algorithm which cannot distinguish the tracing points by walking. DBSCAN is a density-based spatial clustering algorithm. It groups points together that are closely packed. The clusters with different shapes can be discovered and it is not needed to determine the cluster number in advance, and so DBSCAN algorithm is better to mine the potential locations.

## Location prediction method based on the Gaussian Mixture Model(GMM)

### GMM

The prediction model of a user’s location *L*_*r*_ in the time *t* is shown in formula 2.

$$L_{r}=arg\;max_{L_{k}}\;P\left(L_{k}|t\right)\;\left(k=1\ldots n\right)$$ (2)

Here, *L*_*r*_ represents the location with maximum probability value at time *t*.

It is difficult to compute *P*(*L*_*k*_ *|t*) because of the continuity of time *t*, and we transform it to formula 3.

$$P\left(L_{k}|t\right)=\;\frac{P\left(t|L_{k}\right)P\left(L_{k}\right)}{P\left(t\right)}$$ (3)

Here, *P*(*t*) denotes the sign-in probability for the time *t* and *P*(*t|L*_*k*_) denotes the sign-in distribution for location *L*_*k*_ within a day. *P*(*L*_*k*_) denotes the sign-in probability of location *L*_*k*_.

Therefore, the location prediction model is shown in formula 4.

$$L_{r}=arg\;max_{L_{k}}\frac{P\left(t|L_{k}\right)P\left(L_{k}\right)}{P\left(t\right)}$$ (4)

The sign-in time $\hat{t}$ in the dataset is discrete, the GMM is used for modeling *P*(*t*) on the continuous time *t* by formula 5. The Gaussian Mixture Model is a combination of multiple Gaussian distributions. Here, *α*_*i*_ denotes the weight of each Gaussian distribution in GMM.

$$P\left(t\right)=\Sigma_{i=1}^{N}\alpha_{i}Ν\left(t;\mu_{i},\sigma_{i}^{2}\right)\;\left(\alpha_{i}\geq 0,\Sigma_{i=1}^{N}\alpha_{i}=1\right)$$ (5)

Here, $Ν({t;\mu }_{i},\sigma_{i}^{2})$ is the Gaussian distribution in the time *t* and it is computed by formula 6.

$$Ν\left(t;\mu_{i},\sigma_{i}^{2}\right)=\;\frac{1}{\sqrt{2\pi }\sigma_{i}}e^{-\frac{{\left(t-\mu_{i}\right)}^{2}}{2\sigma_{i}^{2}}}$$ (6)

It is similar to compute *P*(*t|L*_*k*_) by the modeling with GMM shown in formula 7.

$$P\left(t^{\left(k\right)}|L_{k}\right)=\;\Sigma_{j=1}^{M_{k}}\beta_{j}Ν\left(t^{\left(k\right)};\mu_{j},\sigma_{j}^{2}\right)\;\left(\beta_{j}\geq 0,\Sigma_{j=1}^{M_{k}}\beta_{j}=1\right)$$ (7)

Here, *t*^*(k)*^ represents the sign-in time for location *L*_*k*_. *P*(*L*_*k*_) is computed by formula 8.

$$P\left(L_{k}\right)=\;\frac{\left|L_{k}\right|}{\sum_{i}\left|L_{i}\right|}$$ (8)

Here, |*L*_*k*_| denotes the tracing point number for location *L*_*k*_ and ∑_*i*_|*L*_*i*_| denotes the total tracing point number for all locations.

Finally, the location prediction model is shown in formula 9.

$$\begin{array}{ll} L_{r} & =\underset{L_{k}}{arg\;max}\frac{\sum_{j=1}^{M_{k}}\beta_{j}Ν\left(t^{\left(k\right)};\mu_{j},\sigma_{j}^{2}\right)\frac{\left|L_{k}\right|}{\sum_{i}\left|L_{i}\right|}}{\sum_{i=1}^{N}\alpha_{i}Ν\left(t;\mu_{i},\sigma_{i}^{2}\right)}\\ & =\frac{arg\;max_{L_{k}}\left|L_{k}\right|\sum_{j=1}^{M_{k}}\beta_{j}Ν\left(t^{\left(k\right)};\mu_{j},\sigma_{j}^{2}\right)}{\sum_{i}\left|L_{i}\right|\sum_{i=1}^{N}\alpha_{i}Ν\left(t;\mu_{i},\sigma_{i}^{2}\right)} \end{array}$$ (9)

### GMM training algorithm

The sign-in frequency for different location *L*_*k*_ is various and so the Gaussian distribution in formula 6 is different. It is needed to adjust dynamically. The algorithm is shown in Table 2.

**Table 2:** GMM training algorithm for different location.

Algorithm 2. GMM Training Algorithm for Different Location
Input: T→=({t0(0),t1(0),t2(0)⋯tN0(0)}{t0(1),t1(1),t2(1)⋯tN1(1)}⋯{t0(M),t1(M),t2(M)⋯tNM(M)}) The time *t* of different tracing point for different *M* locations D: Initial number of Gaussian Model in GMM E: Maximum error value *p_threhold*: Threshold for Gaussian Mixture Coefficient *fix_rate*: Ratio for error modification Output: λ→=(β0→μ0→σ0→β1→μ1→σ1→⋮⋮⋮βM→μM→σM→) Parameter for *M* Gaussian Mixture Models
Begin For $\vec{T_{i}}\;in\;\vec{T}$: Begin d = D //Parameter Initialization e = E // Parameter Initialization do $\left(\begin{array}{ccc} \vec{\beta_{i}} & \vec{\mu_{i}} & \vec{\sigma_{i}} \end{array}\right)$ = EM($\vec{T_{i}}$, d, e)// Solve the *lth* Gaussian Mixture Model by EM Algorithm d = d-1 e = e + *fix_rate*// Update the parameter iteratively while(d >1&& min($\vec{\beta_{i}}$) < *p_threhold*) // Gaussian Model number is more than 1 and min($\vec{\beta_{i}}$) is smaller than the threshold $\vec{\lambda_{i}}\;=\;(\begin{array}{ccc} \vec{\beta_{i}} & \vec{\mu_{i}} & \vec{\sigma_{i}} \end{array})$ End End

Here, EM($\vec{T_{i}}$,d,e) represents that the GMM parameters are achieved by EM algorithm [21]. The Expectation-Maximization (EM) algorithm is an iterative method to find maximum likelihood of parameters in statistical models, where the model depends on unobserved latent variables. In our paper, EM algorithm is used to solve the parameters of Gaussian Mixture Model. If the Gaussian Mixture coefficient is lower than the threshold, the number of Gaussian distribution will be decreased and the Maximum error value will be increased for retraining. Here, we set the p_threshold to 0.01. Although GMM can predict the location by the use of time information, it can’t take into account the context of the trajectory data which limits the prediction performance.

## Location prediction by Markov Model based on the continuous time series(CTS-MM)

Markov model is a stochastic model which assumes that future states depend only on the current state, not on the events that occurred before it. The standard Markov Model cannot give the location prediction based on continuous time series. It only takes into account the context of the trajectory data without the time information. And furthermore, the discrete time series is needed to transform to the continuous time which is simulated by the Gaussian Mixture Distribution with EM algorithm. We put forward the Markov Model based on the continuous time series (CTS-MM), which considers not only the geographic feature in trajectory data but also the time feature.

### CTS-MM

It is known that the user visits location *L*_*i*_ in the time *t* and we want to predict the user’s next location aftertime interval Δ*t*. The model is shown in formula 10.

$$L_{r}=arg\;max_{L_{k}}P(L_{k}|L_{i},t,\Delta t)$$ (10)

**Fig 3:**
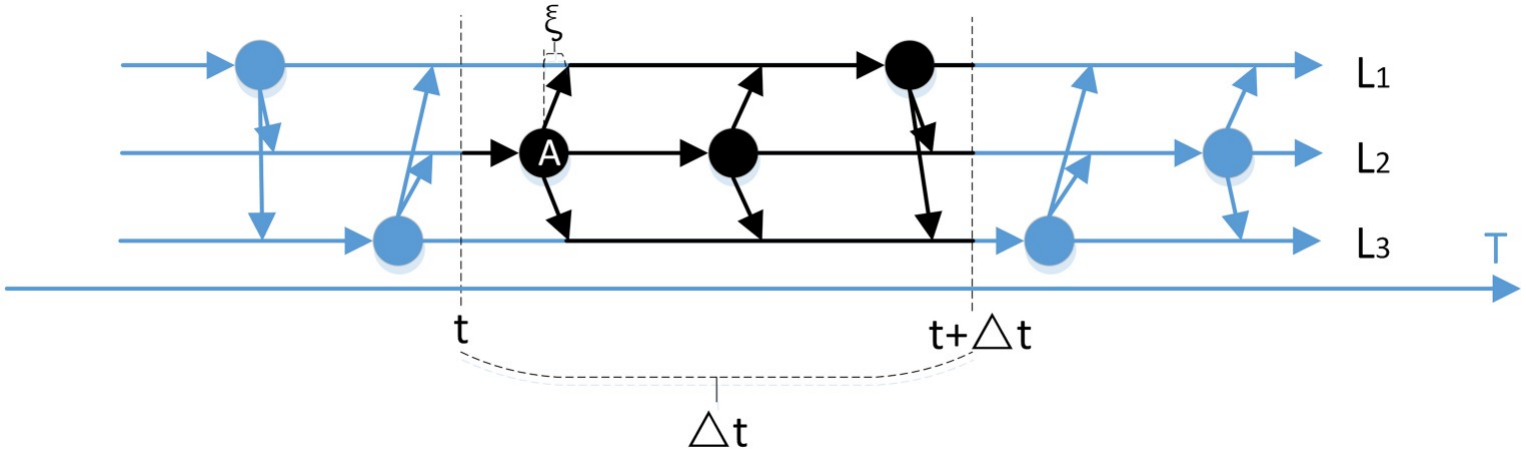
Status transition sample by CTS-MM.

The CTS-MM is used to modeling the user’s visiting sequences, which is shown in Fig 3.

There are three locations *L*_*1*_, *L*_*2*_ and *L*_*3*_ in Fig 3. Each node represents the possible transition time. For example, node *A* denotes a transition time point for location *L*_*2*_ and it can be transferred to location *L*_*1*_ or *L*_*3*_ after time interval *ξ*. For the location *L*_*2*_ at the time *t*, the black arrow and black node in Fig 3 represent the status transition process during the time interval Δ*t*.

The value of *P*(*L*_*k*_ *| L*_*i*_, *t*, Δ*t*) can be calculated with HMM shown in formulas 11 and 12.

$$P\left(L_{k}|L_{i},t,\Delta t\right)=\;\Sigma_{l}P\left(L_{k},I_{l}^{i,k}|L_{i},t,\Delta t\right)$$ (11)

$$\begin{array}{l} P\left(L_{k},I_{l}^{i,k}|L_{i},t,\Delta t\right)=\\ P\left(L_{i}\to L_{a}\right)\times P\left(L_{a}|\Upsilon_{\alpha }^{\left(a\right)}\right)\times P\left(L_{k},I_{l}^{a,k}|L_{a},\Upsilon_{\alpha }^{\left(a\right)},\Delta t-\Upsilon_{\beta }^{\left(k\right)}+\Upsilon_{\alpha }^{\left(a\right)}\right) \end{array}$$ (12)

Here, $I_{l}^{i,k}$ denotes the *lth* trajectory from location *L*_*i*_ to *L*_*k*_ and *L*_*a*_ is the first transferred location after *L*_*i*_ in $I_{l}^{i,k}$. The first item of formula 12 is the transition probability from location *L*_*i*_ to *L*_*a*_. The second item is the conditional probability with formula 3. The third item is a recursion item which represents the transition probability from *L*_*a*_ to *L*_*k*_. The variable ($L_{k},I_{l}^{a,k}|L_{a}$) denotes the transferring status from *L*_*a*_ to *L*_*k*_. The variable ($\Delta t-\Upsilon_{\beta }^{\left(k\right)}+\Upsilon_{\alpha }^{\left(a\right)}$) denotes that the user will change the location from *L*_*a*_ to *L*_*k*_ aftertime interval ($\Delta t-{(\Upsilon }_{\beta }^{\left(k\right)}-\Upsilon_{\alpha }^{\left(a\right)})$).$\Upsilon_{\beta }^{\left(k\right)}$ represents the *β*th transition time for *L*_*k*_ and $\Upsilon_{\alpha }^{\left(a\right)}$ represents the *α*th transition time for *L*_*a*_.

It is needed to get the transition time for each location. $\Upsilon^{\left(i\right)}\;=\;\{t_{1},t_{2},\ldots ,t_{n}\}$ represents thetransition time series for location *L*_*i*_ and they are labeled as the node in Fig 3. The possible transition time is extracted from the training data. Firstly, the location for every sign-in time is recognized. And then select the marginal sign-in time when the location transition occurs. Finally, all of the marginal sign-in time is clustered and the center of each cluster *t*_*1*_, *t*_*2*_, …, *t*_*n*_ is selected as the transition time series. In addition, a random bias *ξ* for *t*_*i*_ is used to simulate the interval of status transition.

### Location prediction algorithm

The location prediction algorithm based on the time series is shown in Table 3, which is implemented by the recursion strategy. For start location *L*_*i*_, predict the next location with the maximum probability after time interval Δ*t*. The array P records the prediction probability for each location.

**Table 3:** Time-Dependent Location Prediction Algorithm (TDLP).

Algorithm 3. TDLP(Time-Dependent Location Prediction Algorithm)
Global Variable:$P_{\;}\;=\;(P(L_{0}L_{i},t,\Delta t),P(L_{1}L_{i},t,\Delta t),\ldots ,P(L_{m}L_{i},t,\Delta t))$ $P_{L_{a}}\;=\;P(L_{a}L_{i},t,\Delta t)$ Input: *L*_*i*_: Start Location *t*_*now*_: Start Time Δ*t*: Transition Time Interval $\Upsilon \;=\;(\Upsilon^{\left(0\right)},\Upsilon^{\left(1\right)},\ldots ,\Upsilon^{\left(m\right)})$: Transition Time Series for Each Location *M* = $\left(\begin{array}{ccc} P(L_{0}L_{0}) & \cdots & P(L_{m}L_{0})\\ \vdots & \ddots & \vdots \\ P(L_{m}L_{0}) & \cdots & P(L_{m}L_{m}) \end{array}\right)$: Location Transition Probability Matrix *P*_*cur*_: The Current Probability with the Initial Value of 1 Output: $argmax\;P_{\;}$
Begin: For 0≤ a≤ m Begin Get the next transition time $\Upsilon_{next}^{\left(a\right)}$ in $\Upsilon^{\left(a\right)}$ relative to *t*_*now*_ if ($\Upsilon_{next}^{\left(a\right)}\;-\;t_{now}\geq \Delta t$) Begin *P*(*L*_*a*_|*L*_*i*_, *t*, Δ*t*) = *P*(*L*_*a*_|*L*_*i*_, *t*, Δ*t*) + *P*_*cur*_ Continue; End else Begin $\;\;\;\;\;\;P_{cur\;}\;=\;P(L_{a},I^{i,a}L_{i},t_{now},\Delta t)$// Compute by formula 12. //Recursion ***TDLP***(*L*a,$\Upsilon_{next}^{\left(a\right)}+\xi$,$\;\Delta t\;-(\Upsilon_{next}^{\left(a\right)}\;-\;t_{now}-\;\xi )\;$,Υ, *M*, *P*_*cur*_) End End End

The probability distribution for each location is calculated recursively. The transition time for location *L*_*a*_ is recorded in the vector $\Upsilon^{\left(a\right)}$, and if there is more time to transfer to next location, the algorithm ***TDLP*** will be implemented recursively. Otherwise, the recursion will be stopped.

Here, *ξ* is a random value used to simulate the transition time interval. It means that user may change location after time interval *ξ*. We give the experiments to set different *ξ* value, and it denotes that the better performance is achieved with *ξ* = 5 minutes, which is shown in the experimental section.

## Experimental analysis

### DataSet

GeoLife, developed by Microsoft, is a location-based social-network project. It enables users to share life experience and build connections among each other by using location history. Furthermore, it contains 182 users’ travel records and total of 17621 trajectories.

**Fig 4:**
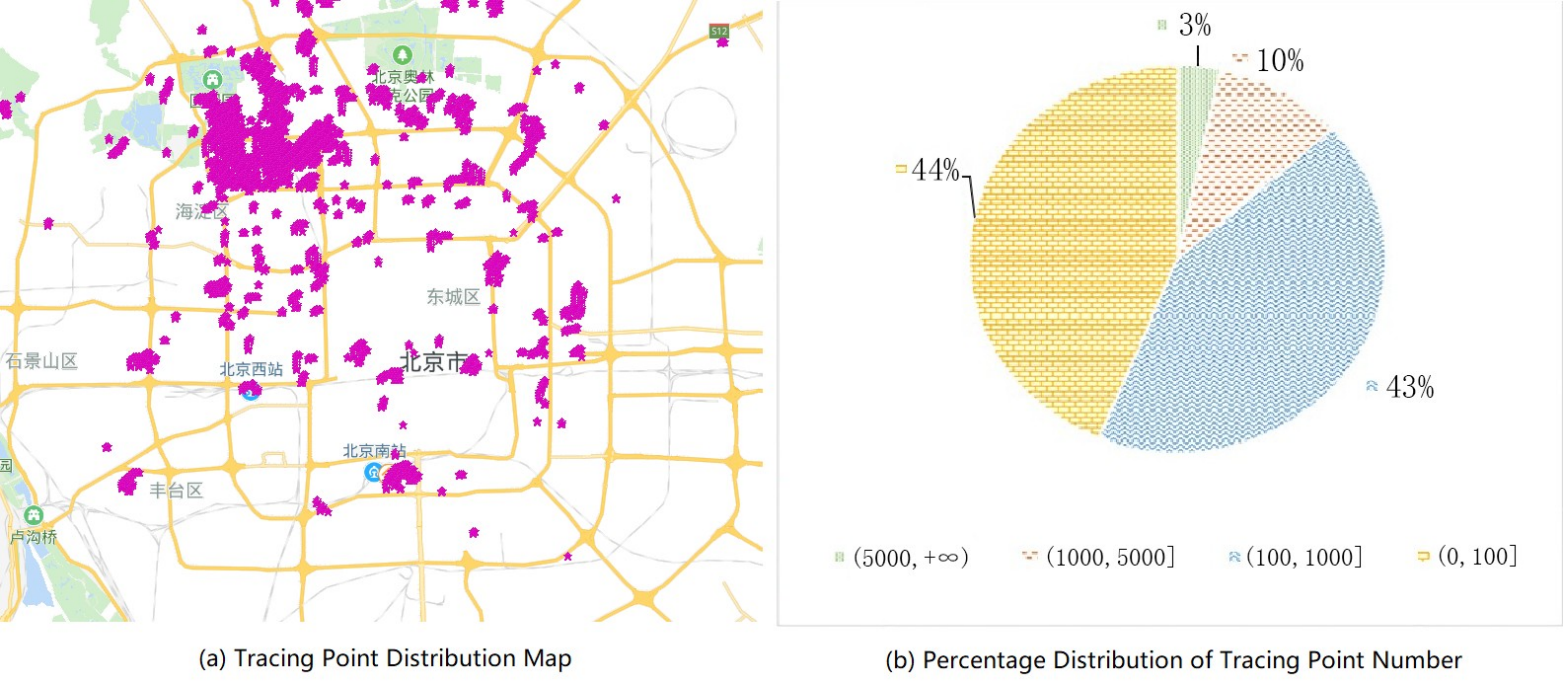
Tracing point distribution on GeoLife.

Most of the tracing points in the dataset are located in Beijing and our experiment also gives the prediction and analysis for location here. The statistical data distribution after filtering and clustering is shown in Fig 4. The parameter of maximum density for DBSCAN clustering algorithm is set to 0.0005 and the minimum size of a cluster is set to 20.

Most locations, around 87%, have only a little tracing points(less than 1000). For total 182 users, average sign-in frequency in these locations is less than 6.

**Fig 5:**
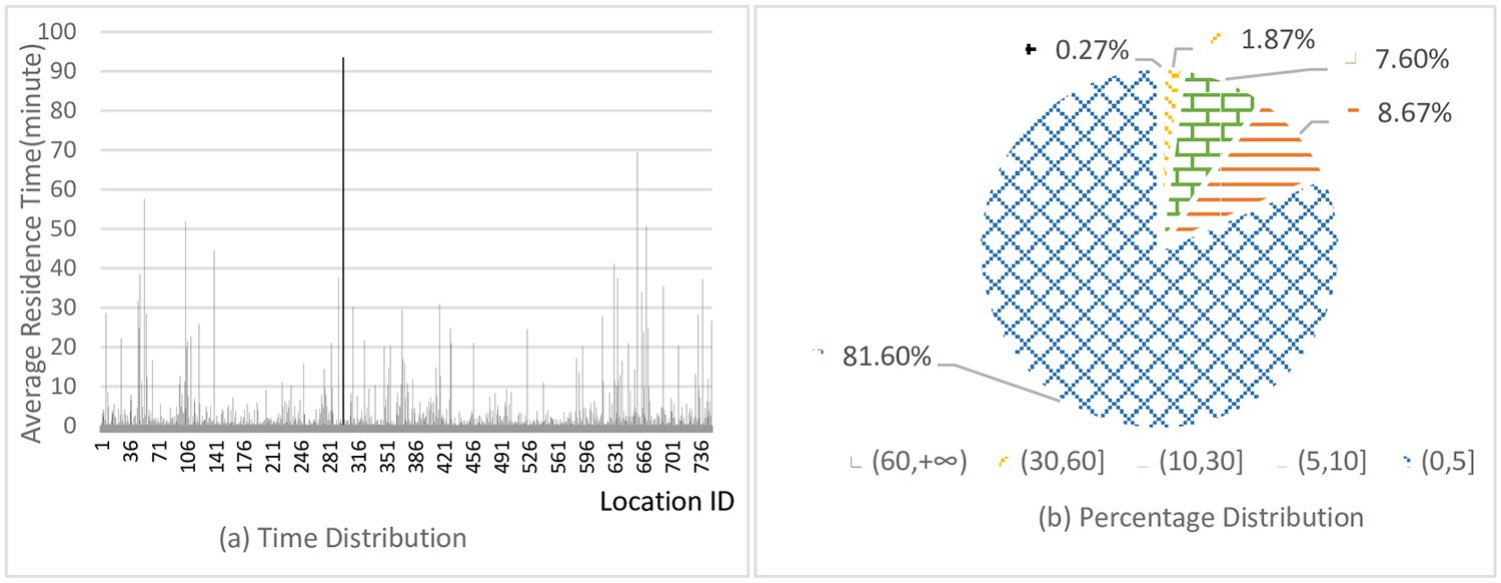
Average residence time distribution.

The average residence time distribution for different locations is shown in Fig 5. It is also found that average residence time is less than 5 minutes for more than 80% locations.

It can be concluded that the distribution of the tracing points is dispersive and user’s moving frequency is relatively fast, which will play an important role in experiment performance.

### Experimental result

**Fig 6:**
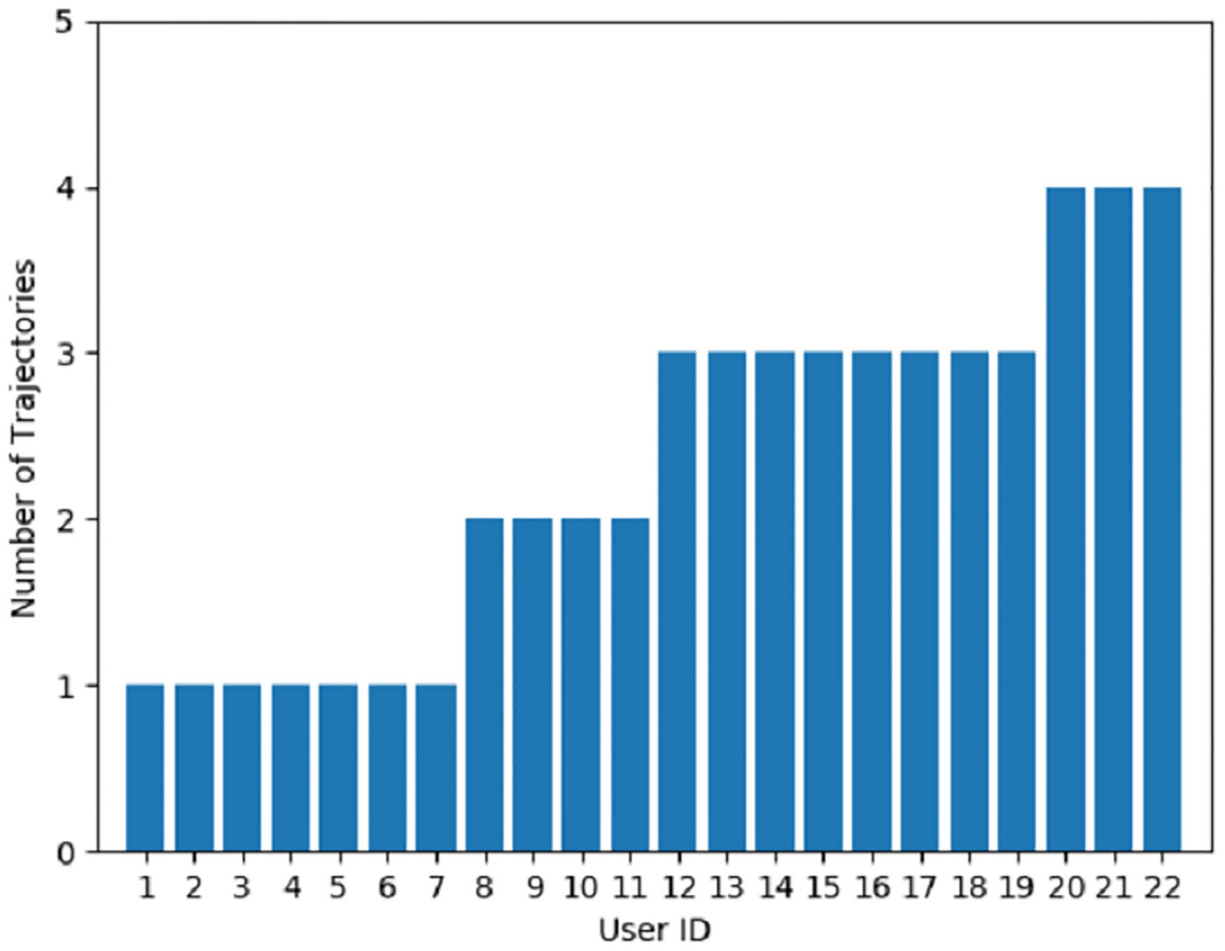
Trajectory distribution of 22 users filtered.

Among the total 182 users, there are 22 users who have trajectory less than 4 and the distribution is shown in Fig 6. They are filtered from the dataset. For the remaining 160 users, we select their trajectory data in adjacent 20 days as training set and the data of next 5 days is used as test set. For users who have trajectory data less than 25 days, we choose 80% of it for training and the rest for testing. The evaluation metric is precision and itis computed by *Precision = A/B*. Here, *A* denotes the number of correct samples by prediction and *B* denotes the total number of samples by prediction.

#### Clustering result for location discovery

**Fig 7:**
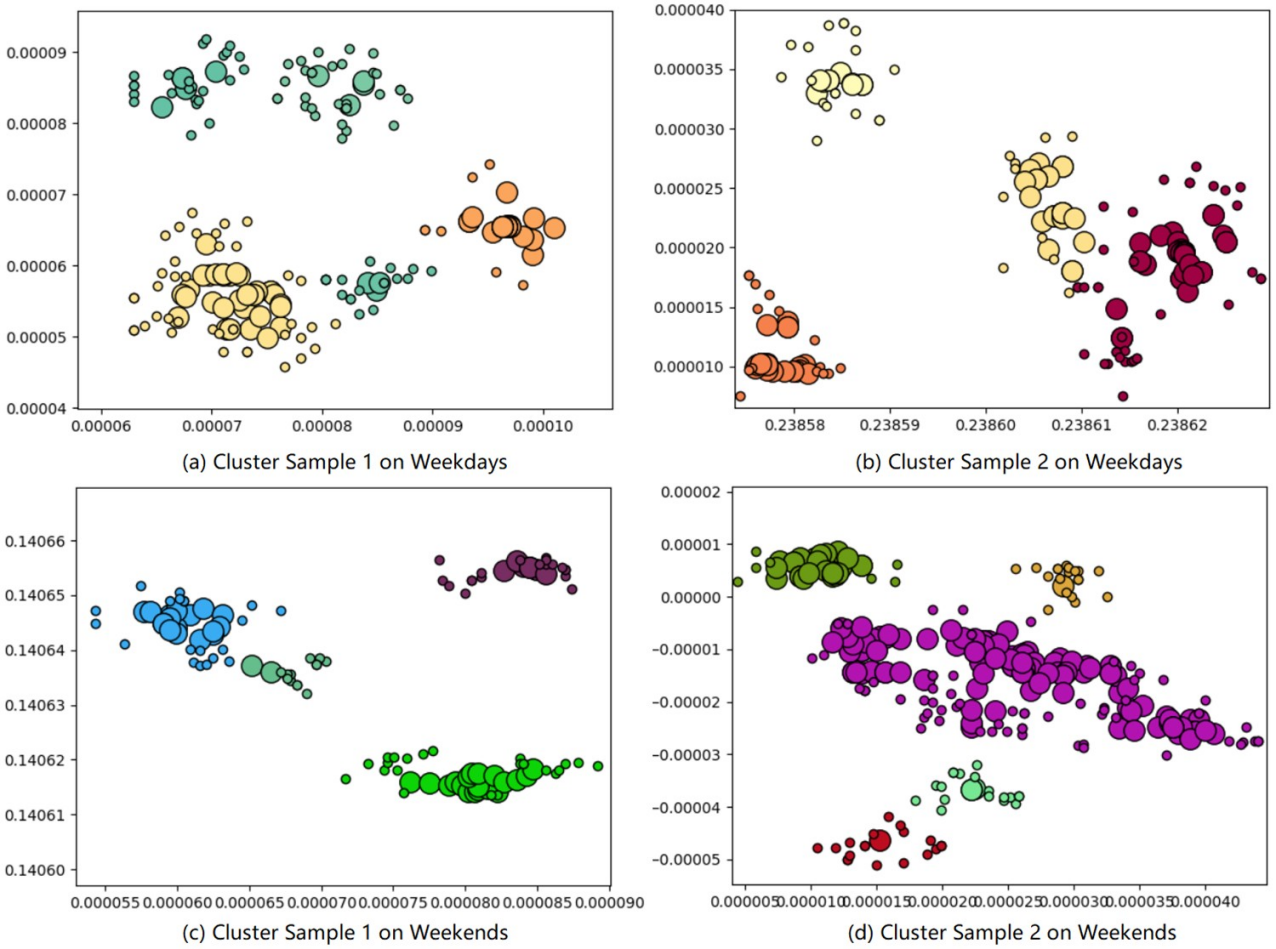
Clustering result sample on weekdays and weekends.

The trajectory distribution has a big difference between weekdays and weekends. We divide the data into two groups for clustering respectively. The sample of clustering results on weekdays and weekends data are shown in Fig 7(a)–7(d).

As it can be seen from Fig 7, there are more tracing points on weekdays than weekends because more activities happened within five weekdays. The DBSCAN algorithm is effective for location clustering, and the boundary of different clusters are clearly in line with reality. Here, the clusters with different color denote different discovered locations.

#### Prediction performance impact by different parameter

- **Impact by different time interval Δ*t***

**Fig 8:**
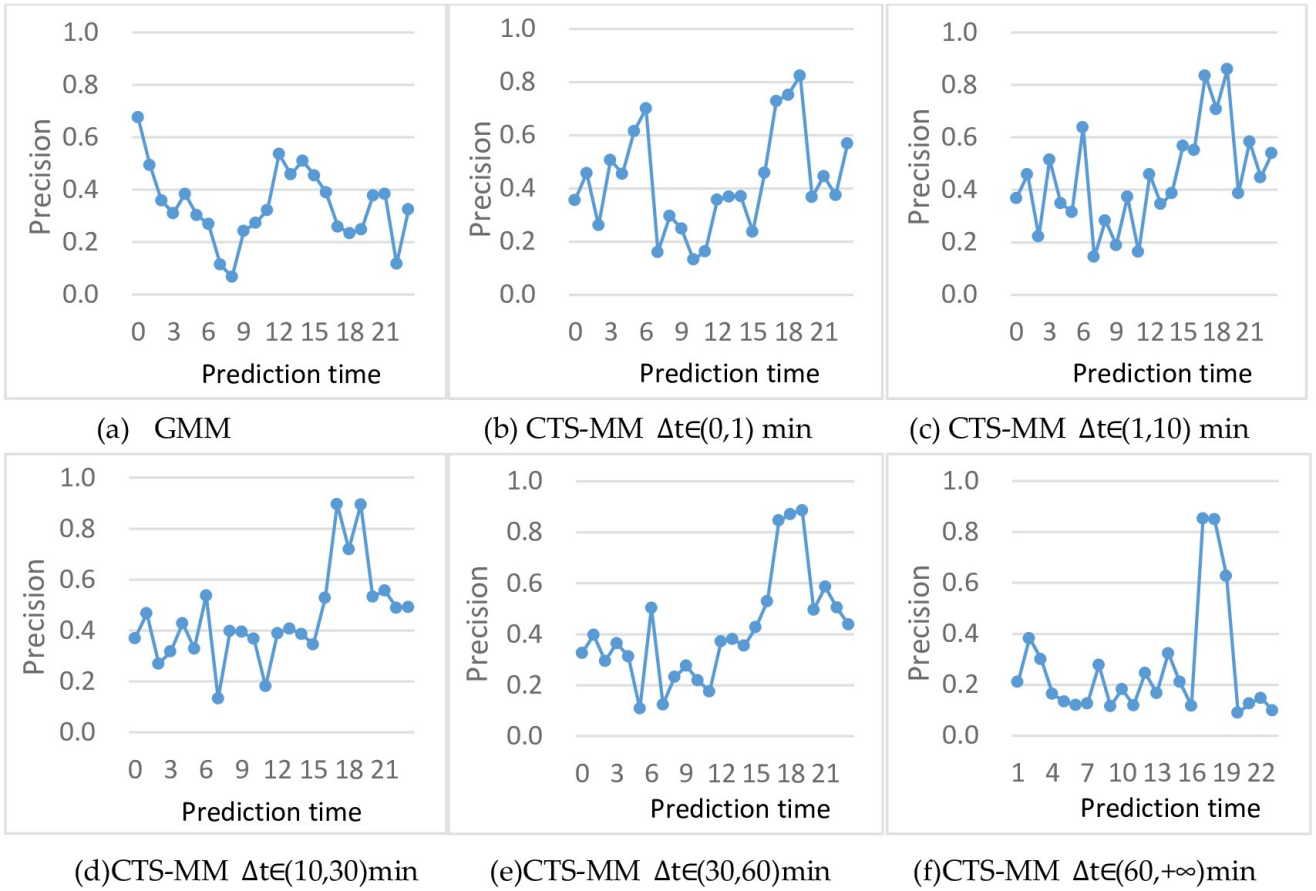
Prediction performance by different time interval Δ*t*.

The evaluation result of location prediction after different time interval Δ*t* is shown in Fig 8.

The prediction precision of GMM is shown in Fig 8(a). For different time interval Δt, the performance of CTS-MM are shown in Fig 8(b)–8(f) separately. It is noticed that the start time of Fig 8(f) is set to 1 o’clock because sign-in behavior across different day is not considered.

We find that the performance varies with time *t* on these two models: GMM and CTS-MM. The precision is higher in early of the morning and lower in other time on GMM, which is shown in Fig 8(a). On the other hand, the prediction performance of CTS-MM shown in Fig 8(b)–8(f) is varied with the increasing of time interval **Δ*t*** which brings too much travel uncertainty. Especially, the precision is higher around 18 o’clock than other time mostly.

- **Impact by different *ξ* value**

The parameter *ξ* mentioned in Table 3 is used to simulate the transition time interval between different locations. We give the experimental results with different *ξ* value in Table 4.

**Table 4:** Prediction precision by different *ξ*.

*ξ*(min)	Δ*t* <1	1<Δ*t*<10	10<Δ*t*<30	30<Δ*t*<60	Δ*t*>60	Average
0	0.416	0.437	0.436	0.394	0.241	0.3848
3	0.421	**0.450**	0.439	0.402	0.248	0.3922
5	**0.425**	0.445	**0.451**	**0.418**	**0.260**	**0.3998**
7	0.425	0.421	0.426	0.394	0.239	0.3810

With different prediction time interval Δ*t*, the prediction performance is better when *ξ* is set to 5 minutes. And the precision gets 0.451 when time interval Δ*t* is set between 10 minutes to 30 minutes.

#### Prediction performance by different user

**Fig 9:**
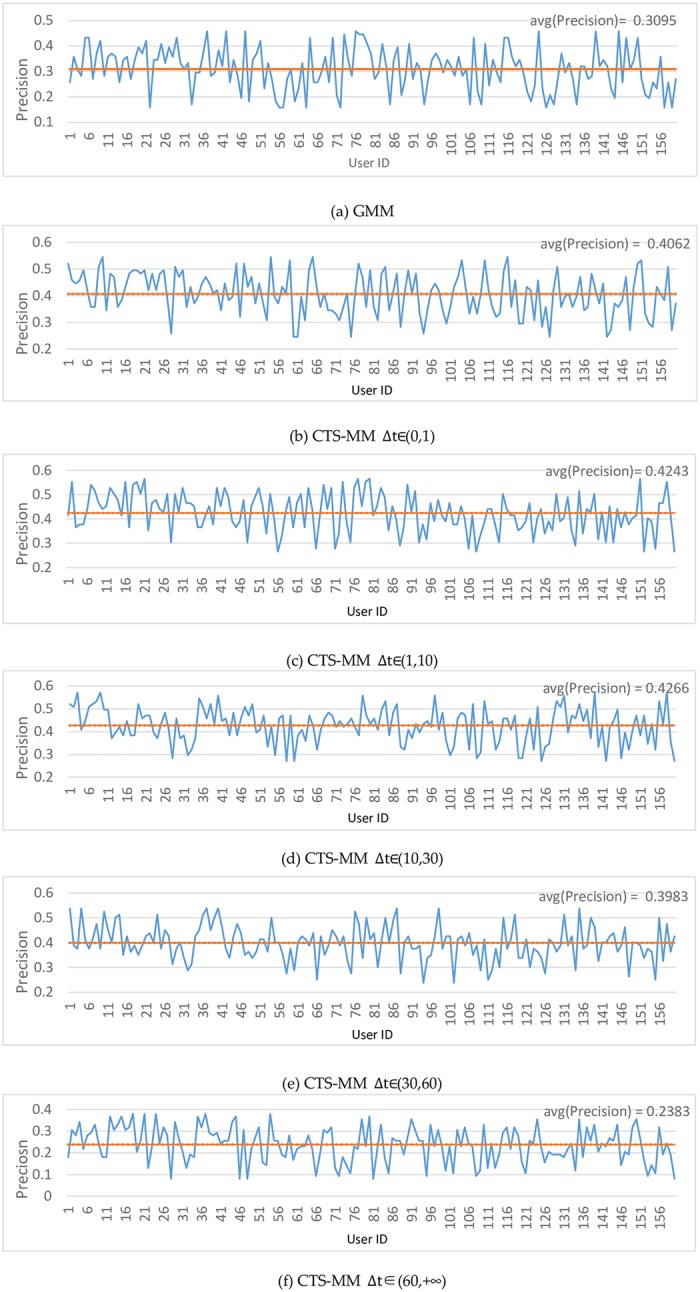
The average precision distribution on different user by different time interval.

We give experiment on different users and the comparison results on both GMM and CTS-MM with different time intervals Δ*t* are shown in Fig 9(a)–9(f).

Compared with the average precision, each user’s prediction performance deviated largely (no more than ±15%). The performance of CTS-MM is improved about 12% with the comparison of GMM when time interval Δ*t* is shorter than one hour. It declines significantly after one hour, which is shown in Fig 9(f). The user’s travel uncertainty increases with longer duration time.

#### Discussion

The prediction performance is evaluated on GeoLife dataset and the average precision is about 43% by CTS-MM. Compared with other related algorithms shown in Table 5, fewer features are used and the precision is higher by CTS-MM.

**Table 5:** Precision of different methods.

	Predict on real time	The best Precision	Features		
			Social Information	Trajectory Information	Sign-in Information
CTS-MM	√	0.4266		√	
GMM	√	0.3095		√	
PST [22] (L = 1)		0.40 ~ 0.41		√	
RCH [23](P = 10)		0.35 ~ 0.40	√	√	√

Our proposed model CTS-MM and GMM can give prediction on continuous time and furthermore CTS-MM performs better than GMM improved by approximately 12% on precision. The trajectory information feature is used for training by these two models. A common problem with related work is the inability to make location predictions on real continuous time. In addition, Song [24]also gives the location prediction by part of the better trajectory with ID number 0 and 17 on GeoLife and the precision varies from 20% to 80%. But the result on all of the trajectory data is not given.

Location prediction on continuous real-time data is a challenging task and our CTS-MM model makes it possible to predict for any time parameter *t*. The real time information is used for training and the model is applied on real time series for prediction, which makes result more reasonable. Our work is implemented on the total dataset of GeoLife and the average precision on different time interval varies from 10% to 90%. Within the time interval of 1 hour, the average prediction precision of all the users varies from 20% to 60%.

We have filtered 22 users who have fewer trajectory from the GeoLife data, and the robustness of our model is not checked on the sparse trajectory data. It may make a difference in prediction performance and the further analysis will be our next step. An effective location prediction can bring a better user experience, such as advertising directed at customers based on their current location.

## Conclusion

With the increasing of user’s location data, the application of location based service becomes more and more popular and it is important to provide superior service for the user. The most common method for location prediction is Markov Model. But it only considers the user’s location movement sequence and it is impossible to give a prediction related to the time information. We put forward a new method by Markov Model based on the Continuous Time Series(CTS-MM). The Bayes model and GMM are used for modeling the posterior probability of the location with continuous time series. The discrete status sequence of the HMM is changed to continuous sequence, which enables the model to predict the location in different real time. The experimental results on GeoLife data denote that the proposed model achieves a higher precision than traditional methods. The distribution of the tracing points on GeoLife is dispersive and the user’s moving frequency is relatively fast, which makes the prediction task more challenging. Specially, with the increasing of time interval Δ*t*, the precision will decline because it brings much travel uncertainty.

In future work, other models will be considered to improve prediction performance. At the same time, some other effective information, such as sign-in data and user data, will be used for assistance.

## Supporting information

S1 File Geolife Trajectories 1.3.part01.rar. This file is part of the Microsoft dataset.
(RAR)

S2 File Geolife Trajectories 1.3.part02.rar. This file is part of the Microsoft dataset.
(RAR)
